# Characterisation of Extracellular Vesicles from Equine Mesenchymal Stem Cells

**DOI:** 10.3390/ijms23105858

**Published:** 2022-05-23

**Authors:** Robert Soukup, Iris Gerner, Sinan Gültekin, Hayeon Baik, Johannes Oesterreicher, Johannes Grillari, Florien Jenner

**Affiliations:** 1 VETERM, Equine Surgery Unit, Department for Companion Animals and Horses, Vetmeduni, 1210 Vienna, Austria; robert.soukup@vetmeduni.ac.at (R.S.); iris.gerner@vetmeduni.ac.at (I.G.); sinan.gueltekin@vetmeduni.ac.at (S.G.); hayeon.baik@vetmeduni.ac.at (H.B.); 2 Austrian Cluster for Tissue Regeneration, 1200 Vienna, Austria; 3 Ludwig Boltzmann Institute for Traumatology, The Research Center in Cooperation with AUVA, 1200 Vienna, Austria; johannes.oesterreicher@trauma.lbg.ac.at; 4 Institute of Molecular Biotechnology, University of Natural Resources and Life Sciences, 1090 Vienna, Austria

**Keywords:** extracellular vesicles, equine, mesenchymal stem cell, EV isolation, EV characterisation

## Abstract

Extracellular vesicles (EVs) are nanosized lipid bilayer-encapsulated particles secreted by virtually all cell types. EVs play an essential role in cellular crosstalk in health and disease. The cellular origin of EVs determines their composition and potential therapeutic effect. Mesenchymal stem/stromal cell (MSC)-derived EVs have shown a comparable therapeutic potential to their donor cells, making them a promising tool for regenerative medicine. The therapeutic application of EVs circumvents some safety concerns associated with the transplantation of viable, replicating cells and facilitates the quality-controlled production as a ready-to-go, off-the-shelf biological therapy. Recently, the International Society for Extracellular Vesicles (ISEV) suggested a set of minimal biochemical, biophysical and functional standards to define extracellular vesicles and their functions to improve standardisation in EV research. However, nonstandardised EV isolation methods and the limited availability of cross-reacting markers for most animal species restrict the application of these standards in the veterinary field and, therefore, the species comparability and standardisation of animal experiments. In this study, EVs were isolated from equine bone-marrow-derived MSCs using two different isolation methods, stepwise ultracentrifugation and size exclusion chromatography, and minimal experimental requirements for equine EVs were established and validated. Equine EVs were characterised using a nanotracking analysis, fluorescence-triggered flow cytometry, Western blot and transelectron microscopy. Based on the ISEV standards, minimal criteria for defining equine EVs are suggested as a baseline to allow the comparison of EV preparations obtained by different laboratories.

## 1. Introduction

Mesenchymal stem/stromal cells (MSCs) can be isolated from various sources such as adipose tissue, bone marrow, umbilical cord matrix, cord blood, blood or synovial fluid and have shown promising therapeutic potential for the treatment of musculoskeletal disorders such as tendinopathy and osteoarthritis in both human and equine patients [1,2,3,4,5,6,7,8,9,10,11]. However, the transplantation of viable replicating cells presents safety concerns and limitations regarding standardisation, quality control and donor-dependent therapeutic potential [12,13,14,15]. MSCs exert their therapeutic effect not by engraftment and differentiation but predominantly by secreting a wide range of bioactive molecules such as cytokines, enzymes and growth factors, collectively referred to as secretome, which induce and support endogenous regeneration and modulate the immune response. The secretome, consisting of soluble and extracellular vesicle (EV) bound proteins, lipids, and nucleic acids, has proven to have equivalent therapeutic effects as the parental cells, providing the opportunity to develop cell-free regenerative therapies [16,17,18,19,20]. EVs alone can also achieve this regenerative potential, which offers additional advantages over the use of the whole secretome, as they protect their encapsulated or associated contents from degradation and can be stored without potentially toxic cryopreservatives [21,22,23,24,25].

EVs are a heterogeneous population of nanosized membrane-encapsulated particles secreted by cells into the extracellular environment. They are formed by a phospholipid bilayer originating from their parent cell and encapsulate nucleic acids, lipids and proteins [26] EVs contribute to cell-to-cell signalling in health, ageing and disease and are a promising tool for therapeutic and diagnostic applications [27,28,29,30,31,32,33]. Various subsets of EVs have been categorised according to their diameter as apoptotic bodies (>1000 nm), microvesicles (100–1000 nm) and exosomes (30–150 nm) [34]. The latter two are commonly referred to as extracellular vesicles. Due to their overlapping size, EVs can be further distinguished by their biogenesis pathways, their cell of origin and their cargo [35]. 

The functionality and hence therapeutic efficacy of EVs is determined by their cargo, which depends on the origin and activation status of the producer cell, and their surface and transmembrane molecules that govern target specificity and EV uptake by recipient cells [36,37,38,39,40,41,42,43,44,45,46,47,48,49,50]. The resulting heterogeneity of EVs in both content and functionality necessitates and impedes standardised, well-defined EV manufacturing processes to determine their therapeutic efficacy and achieve reliable therapeutic EV dosing in preclinical and clinical settings [38,39,40,41,42,43,44,45,46,47,48,49,50]. Unfortunately, the current lack of standardised isolation protocols and characterisation strategies limits the comparability and reproducibility of results obtained by different laboratories [42]. The International Society for Extracellular Vesicles (ISEV) published a position paper to provide minimal criteria (MISEV criteria) for EV isolation and characterisation to better exchange EV data and counteract methodological inconsistency when working with EVs [51]. Standardised purification and analytical methods will facilitate the discovery of functional heterogeneity and the production of EV-based regenerative therapies.

The isolation strategy for EV preparations is dependent on the volume and type of fluid from which the EVs are separated (cell culture supernatant, biological fluids such as blood plasma/serum, etc.) and needs careful consideration since it directly affects the isolated EV population and EV purity [38,39,40,41,42,43,44,45,46,47,48,49,50]. According to a survey from 2019, the most commonly used method to isolate EVs is differential ultracentrifugation (UC), followed by size exclusion chromatography (SEC) [52]. UC separates particles by sedimentation using different centrifugation forces and durations [52]. The EV extraction efficacy of this method is determined by a combination of acceleration, rotor type, viscosity and duration of centrifugation [46,47,48,49]. In contrast, SEC uses the biofluid as a mobile phase and the porous resin particles as a stationary phase to isolate particles of the desired size. Smaller particles enter the stationary phase’s pores and elute later, while particles bigger than the isolation range flow around the resin and elute earlier from the column [38,39,40,50].

Each method leads to different yields and purities of EVs and is accompanied by certain advantages and disadvantages such as time efficiency, costs and sample volume [40,41,42]. Most researchers adopt these two methods with slight differences, which, again, leads to problems concerning the reproducibility of experiments, especially if publications lack proper documentation.

The varying degree of EV population heterogeneity and purity introduced by the choice of isolation methods also confounds the measurement of EV purity and content, which are essential determinants for EV dosing. Currently, protein concentration and particle count are the most commonly used dosing strategies; however, different EV isolation methods can, due to coisolating contaminating proteins, yield samples with up to an eightfold difference in protein content relative to particle number from the same source material. Moreover, other parameters used to quantify EVs, such as total lipid abundance, total RNA or the presence of specific molecules, do not perfectly correlate with the actual EV number, emphasising the need for in-depth reporting of the isolation and quantification methods used in each study and for the standardisation of EV production procedures [51,53,54].

The potential benefits of administered EVs in horses have been shown in various studies [52,55,56]. However, the comparisons of different protocols for equine EV work are still vastly missing, which is also attributed to the lack of working antibodies. Therefore, in this study, we characterised EVs isolated from equine bone-marrow-derived MSCs (bmMSCs) following the ISEV guidelines and compared the two most common isolation methods, UC and SEC, with respect to EV yield, purity, cost and time efficacy, the complexity of the protocol and the need for specialised equipment. Differences in surface marker stability, size uniformity and number of particles were assessed by a Western blot, nanotracking analysis (NTA) and fluorescence-triggered flow cytometry (FT-FC).

## 2. Results

### 2.1. Cells Isolated from Equine Bone Marrow Show Distinct MSCs Characteristics

**Figure 1:**
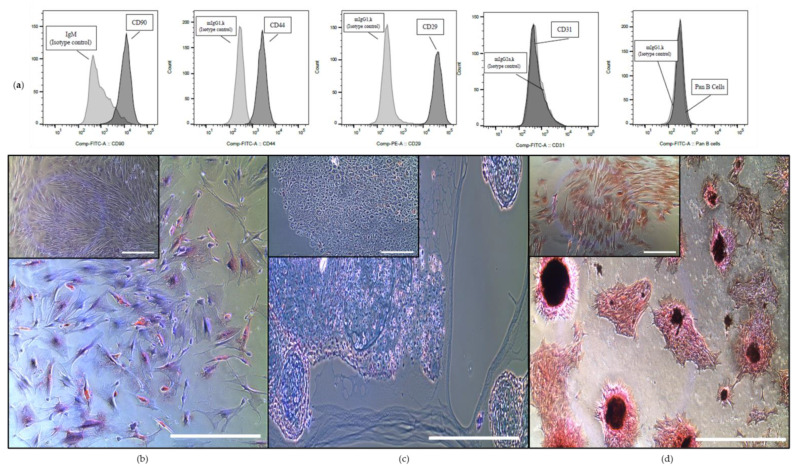
Characterisation of the equine bone-marrow-derived cells showing trilineage differentiation capacity and canonical surface marker expression of equine MSCs. (**a**) Bone-marrow-derived cells were stained with the indicated cell surface antigens (dark grey plots) or Immunoglobulin (Ig) isotype controls (light grey plots) and analysed by flow cytometry (one representative FT-FC experiment is shown). Cells stained positive for CD90, CD44 and CD29 and negative for CD31 and Pan B, as well as IgG isotype controls. Displayed on the x-axis is either Phycoerythrin (PE) or Fluorescein isothiocyanate (FITC) conjugated to one the previous mentioned antibodies. (**b**–**d**) Bone-marrow-derived cells showing trilineage differentiation into the adipogenic ((**b**) stained with Oil red O, scale bar: 400 µm), chondrogenic ((**c**) stained with Alcian blue, scale bar: 400 µm and 100 µm for the control) and osteogenic ((**d**) stained with von Kossa stain, scale bar: 400 µm) lineage. The corresponding controls (cells grown in expansion medium) are shown in the insert in the top left corner of each micrograph.

The cells isolated from the bone marrow of the three donor horses were plastic adherent and positive for the MSC markers CD90, CD44 and CD29 and negative for CD31 and Pan B (Figure 1a). The isotype controls were negative. Furthermore, the bone-marrow-derived cells showed adipogenic (Figure 1b), chondrogenic (Figure 1c) and osteogenic (Figure 1d) trilineage differentiation potential after three weeks in culture (Figure 1). The control samples, which were cultured in standard DMEM media with 10% FCS, showed no indication of differentiation.

### 2.2. EV Isolation Strategy

**Figure 2:**
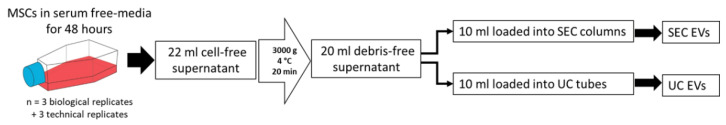
Illustration of the experimental setup: MSCs derived from the bone marrow of 3 equine donors were cultured in serum-free DMEM for 48 h (*n* = 3 biological replicates, plus 3 technical replicates per donor). Then, 22 mL of cell-free supernatant was collected from 8 × 10^6^ cells per replicate and centrifuged in a 50 mL falcon tube with 3000× *g* for 20 min at 4 °C. In total, 20 mL of debris-free supernatant was recovered. Half of it was used to isolate EVs by the SEC columns, and the other half was filled into UC tubes.

EVs were isolated from equine bone-marrow-derived MSCs conditioned media (8 × 10^6^ cells per replicate) of all three donors and three independent culture flasks using either SEC or UC. After 48 h (seeding density at 0 h: 4 × 10^6^ cells per T175 flask), 22 mL serum-free medium yielded 20 mL debris-free supernatant following centrifugation, which could then be further processed in 10 mL aliquots using SEC and UC to isolate EVs (Figure 2).

### 2.3. Nanotracking Analysis Shows Significantly Higher EV Yield by UC Compared to SEC

**Figure 3:**
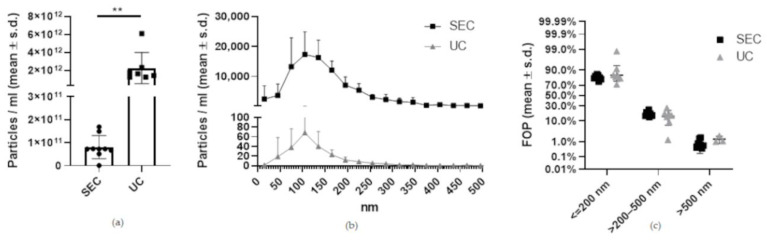
Particle number and size distribution of equine MSC-EVs based on NTA measurements. (**a**) Significantly more particles per ml were found in the UC isolates (** *p* = 0.0021). Black dots correspond to the SEC isolated samples and black squares to the UC isolated samples. (**b**) However, both methods resulted in a similar size distribution pattern, with most particles present below 200 nm. (**c**) Statistical analysis of the size distribution of particles identified by NTA analysis showed no significant differences of particles isolated using the UC or SEC.

The EV yield measured by NTA was significantly different (*p* < 0.0021) between the two isolation methods with UC yielding 27.7 times more EVs from 10 mL SN (2.24× 10^12^ ± 1.6 × 10^12^ (mean ± s.d.) particles/mL) than SEC (8.08 × 10^10^ ± 4.73× 10^10^ particles/mL) (Figure 3a). The size distribution between the SEC and UC was similar with no statistically significant differences (Figure 3b,c). Specifically, in the SEC isolate, the frequency of parents (FOP) of the particles ≤200 nm was 81.7% ± 2.9%, the FOP >200–500 nm was 17.5% ± 2.9% and the FOP >500 nm was 0.7% ± 0.5%. In the UC isolates, the FOP of the particles ≤200 nm 84.8% ± 7.9%, FOP >200–500 nm was 14.6% ± 7.3% and the FOP >500 nm was 1.4% ± 0.5%.

### 2.4. Fluorescence-Triggered Flow Cytometry Shows a Significantly Higher Yield of CMG and CD81-Positive Particles by UC Compared to SEC

**Figure 4:**
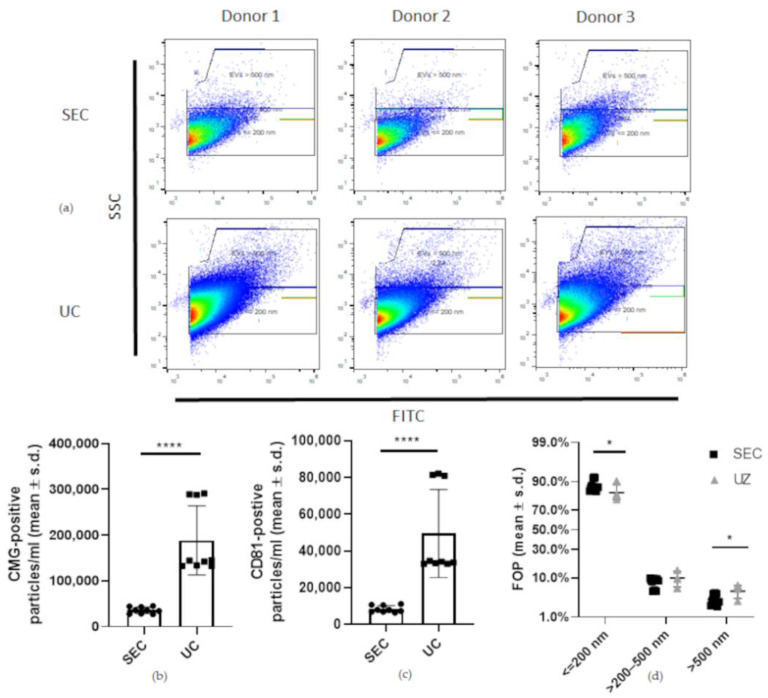
Flow-cytometry-based characterisation of equine MSC-EVs. (**a**) Representative image of fluorescence-triggered flow cytometry analysis of EV isolates enriched by either SEC (**top row**) or UC (**bottom row**). Data were collected by measuring the CD81 signal and CMG (CellMask Green) signal separately (n = 3 technical replicates). On the *y*-axis is the side scattering (SSC) and on the *x*-axis is FITC. (**b**) Significantly (**** *p* < 0.0001) more particles per millilitre stained positive for CMG in the UC isolates (188,752 ± 71,241 particles/mL) compared to the SEC isolates (35,811 ± 6729 particles/mL) (**c**). Significantly (**** *p* < 0.0001) more particles per millilitre stained positive for CD81 in the UC isolates (49,616 ± 22,588) compared to SEC (8470 ± 1751) (**d**). The size distribution of particles ≤200 nm (* *p* = 0.0446) and >500 nm (* *p* = 0.0105) identified by the FT-FC analysis was statistically significantly different between the two isolation methods.

Fluorescence-triggered flow cytometry (FT-FC) confirmed the presence of EVs in isolates obtained by both UC and SEC (Figure 4a). UC isolated significantly (*p* < 0.0001) more particles labelled by the lipid membrane dye cell mask green (CMG) (188,752 ± 71,241 (mean ± s.d.) particles/mL) and EV-specific CD81 positive particles (49,616 ± 22,588) than SEC (CMG: 35,811 ± 6729, CD81: 8470 ± 1751 particles/mL) (Figure 4b,c). The yield obtained with UC was thus 5.3-fold higher than SEC for CMG positive particles and 5.85-fold higher for CD81 positive particles. Particle counts based on CMG and CD81 labelling correlated strongly (r = 0.9959, *p* < 0.0001), with 25.9% of CMG-labelled particles staining positive for CD81.

The differences in size distribution between the two isolation methods were subtle but statistically significant for CMG-positive particles ≤200 nm (*p* = 0.0446) and >500 nm (*p* = 0.0105) but not for >200–500 nm (*p* = 0.1413) with SEC isolates yielding more particles in the desired size range (≤200 nm) and fewer larger particles (Figure 4d). Specifically, in the SEC isolates, the FOP of the CMG positive particles ≤200 nm was 88% ± 2.8%, the FOP >200–500 nm was 7.8% ± 1.9% and the FOP >500 nm was 3.1% ± 1.0%. In the UC isolates, the FOP of the CMG positive particles ≤200 nm was 83.8% ± 4.6%, the FOP >200–500 nm was 9.9% ± 3.3% and the FOP >500 nm 5.1% ± 1.6%.

Based on these measurements, 318 mL of SN is required to produce 1 × 10^6^ CMG positive particles ≤200 nm using SEC and 63.8 mL of SN using the UC methods.

FT-FC (CMG-positive) and the NTA measurements of total particle numbers (r = 0.4987, *p* = 0.0493) and size distribution relative to FOP (r = 0.9835, *p* < 0.0001) significantly correlated. However, FT-FC (CD81-positive) and the NTA measurements of total particle numbers (r = 0.439, *p* = 0.0889) did not correlate.

### 2.5. Western Blot Shows Positive Signals for Two Tetraspanins to Which Cross-Species Reactive Antibodies Are Available

**Figure 5:**
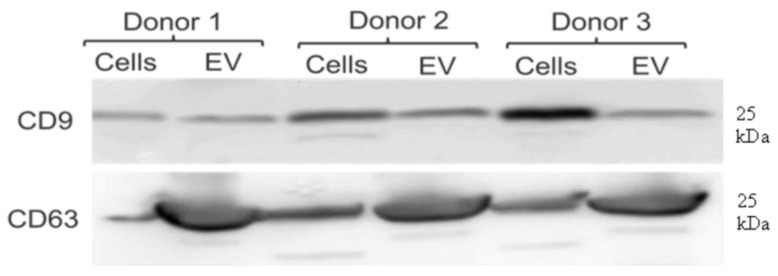
Western blot of equine MSC cell isolates and UC isolated EVs: All isolates from all donors were positive for CD9 and CD63 in the whole-cell lysates and their respective EV isolates.

Whole-cell lysates and the EV populations enriched by UC were analysed using a Western blot to check for the presence of tetraspanins using human anti-CD9 and anti-CD63 antibodies. Indeed, the equine MSCs and isolated EVs showed positive signals for these two tetraspanins, which are markers of membrane proteins commonly found across all cell and EV types (Figure 5).

### 2.6. Transelectron Microscopy (TEM) Shows Particle Morphology

**Figure 6:**
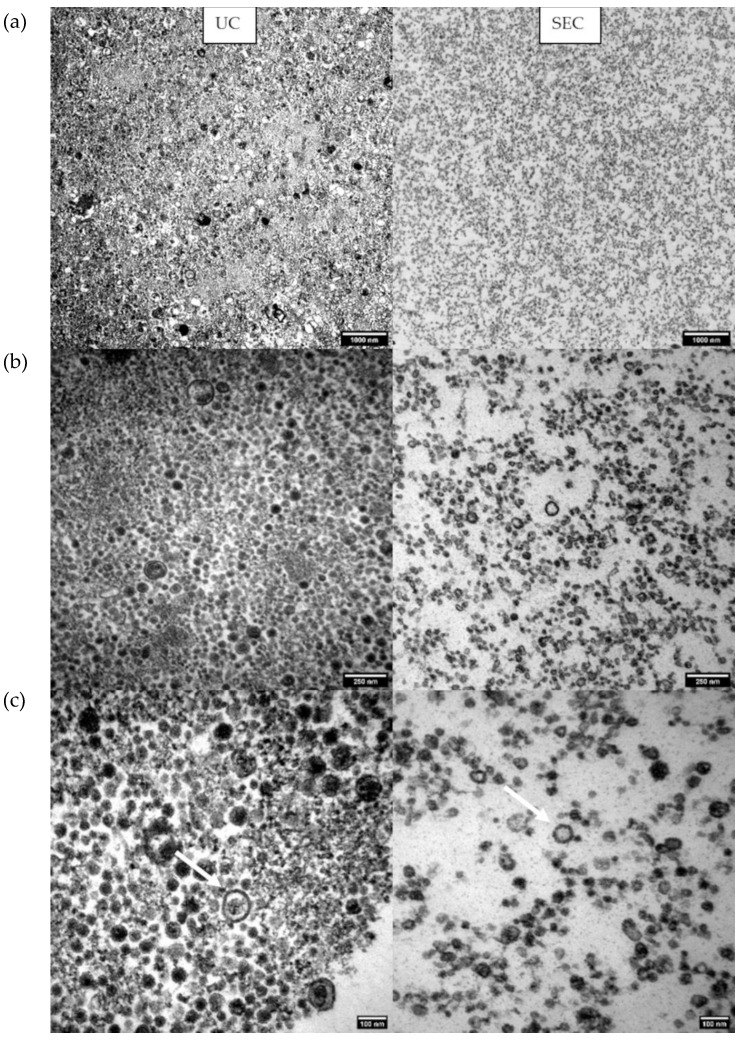
Transelectron Microscopy (TEM) images of the UC EV isolates in the left panels and the SEC EV isolates in the right panels taken at decreasing (top to bottom) magnifications: (**a**) 12,000× magnification gives an overview of the different sizes of the particles with a 1000 nm scale bar; (**b**) 50,000× magnification shows the different electron densities by different grey scales; (**c**) 85,000× magnification shows clearly the EVs identifiable by their lipid bilayer (arrows). In the UC isolates, the size distribution of the EVs is heterogeneous, ranging from large particle agglomerates to small EVs. In contrast, in the SEC isolates, particles are more uniformly sized without agglomerates.

TEM demonstrated particles sized 35 nm–350 nm surrounded by a lipid bilayer with a variable electron density cargo content, thus confirming the presence of EVs (Figure 6). In the UC isolates, larger vesicles and agglomerates were evident, while in the SEC isolates, vesicles were more homogenously sized, consistent with the size distribution measured by FT-FC (Figure 6). In addition, the potential variation of cargo in the EVs was shown by different electron densities evidenced by different grey scales in the TEM pictures (Figure 6).

## 3. Discussion

To facilitate the use of EVs as therapeutics in veterinary medicine, gold standards for EV isolation, characterisation and quality control are urgently required.

EV samples have complex inherent biophysical and biochemical properties such as size, mass density, shape, charge or antigen exposure [57]. Depending on the EV source such as blood, plasma or cell culture supernatant, and intended application, such as a single EV analysis, functional assays or therapeutic administration, several factors need to be considered before choosing an appropriate isolation method or a combination of methods to adapt to the specific needs of EV research [53,54,58]. As the composition of the enriched EVs will differ depending on the isolation protocol used, standardized assays evaluating the yield and size distribution are mandatory before proceeding to ensure reproducibility and facilitate the interpretation of the therapeutic efficacy and scientific community-wide data comparison. In this study, we suggested MISEV criteria for the isolation and characterisation of equine MSC-derived EVs.

EVs were successfully and reproducibly isolated from equine bone-marrow-derived MSCs using both UC and SEC, facilitating a broad comparison of UC and SEC. The MSCs were cultured in serum-free medium for 48 h to exclude possible interferences with serum during the isolation process, as EVs present in the FCS or other serum types used for cell culture can significantly affect the perceived yield of EVs and exert downstream effects, including both therapeutic as well as (xenogeneic) inflammatory effects [59].

UC produced more particles of the desired size range (≤200 nm) per millilitre of supernatant than SEC due to its higher isolation efficacy. The size cutoffs for FT-FC were chosen based on a gating with fluorescence-labelled silica beads of defined sizes, which most closely approximated the dimensions of the different EV subtypes (small ≤200 nm, intermediate 200–500 nm and large >500 nm) [60]. The beads served as a size reference for the distinction of the EV subtypes to separate exosomes (the particles of interest for this study) from the other subtypes because exosomes (30–150 nm) have a defined mode of biogenesis and are highly interesting for regenerative applications, because they have been shown to play essential roles in intercellular communication [61,62]. EVs above that size are less homogenous in origin, e.g., particles above 500 nm are either apoptotic bodies, conglomerates of proteins or other contaminants which are not desired and are characteristic of poor EV isolates [51]. More particles stained positive for CMG than CD81 because this dye incorporated into the lipid bilayer, which was present in all EVs, rather than detecting a tetraspanin like CD81, which was enriched on EVs and not obligatorily present on all EVs [63,64].

The advantages of the UC method include a higher yield and a broad particle size isolation range. However, EVs isolated through UC reportedly have diminished functional capacity compared to EVs isolated using SEC, potentially due to prolonged exposure to high centrifugal forces [38,41]. Furthermore, UC requires more delicate sample handling, complex equipment and hence more experience to obtain a good EV yield than with SEC. In addition, variable UC protocols and parameters involved in the process of EV isolation, such as rotor type, UC-tube quality, centrifugation speed, acceleration, and deceleration, which can be individually adjusted to improve the purity and yield of the isolates, may lead to difficulties with respect to reproducibility and variation between laboratories. Other disadvantages of this method include the potential contamination with exosomal aggregates, which reduces purity and may affect the accuracy of EV quantification and the price of the equipment [48,65,66,67]. 

The main advantages of SEC are the simpler technique and the higher purity of the obtained EVs with minor artefacts such as EV aggregates, which facilitate clinical applicability [49,50].

To translate EV-based therapeutics to clinical practice, their quality, safety, and efficacy must be demonstrated, as required for any medicinal product. EV purity is of critical importance for evaluating safety, dosage, potency and efficacy. Specifically, EV purity is crucial to ensure that any observed biological effects are due to EV cargoes and not copurified contaminants, and that EV preparations are free of contaminating proteins and nucleic acids that could have a negative impact upon clinical administration. Copurified protein aggregates can also artificially increase yield measurements and confound EV quantification. A reliable quantification of EVs is essential to ensure batch-to-batch reliability and reproducibility between applications, identify effective dose ranges for different therapeutic EV preparations and develop dependable dosing strategies.

The two main EV enumeration procedures currently in use are particle number and size distribution, as assessed by NTA and protein concentration. However, it is well established that the protein concentration does not correlate with particle number readout [49,51,53,68]. A recent metareview looking at dosing regimens found that more than 50% of all studies quantified EVs in total amount of protein and 18% utilised NTA, whereas 21% of the studies did not quantify EVs in any way [47]. As the heterogeneity of EVs and presence of lipoproteins and protein aggregates confound current EV quantification methods, future strategies to standardize EV dosing may also focus on qualitative aspects such as the quantity of known effector molecules or EV potency units based on standardised in vitro potency assays [49]. Therefore, in this study, we characterised equine EVs based on the MISEV criteria using a Western blot, FT-FC, NTA measurements and TEM to address the limited availability of cross-reacting markers for horses. The isolated particles stained positive for CD9 and CD63 as demonstrated by a Western blot and for CD81 using FT-FC [57,69,70]. FT-FC, NTA and TEM confirmed the EV-appropriate size range (between 30 and 150 nm) and TEM the presence of the characteristic lipid bilayers surrounding EVs [71,72].

The absence of validated antibodies is a considerable obstacle in working with equine cells. Although the horse and the human genome are highly conserved, the binding of human antibodies to equine targets is in most cases not effective [59,60]. BLASTED protein sequences of standard EV markers showed a homology of 84% for CD63, 92% for CD9, 98% for TSG101 and 97% for CD81 between horses and humans. We tested available antibodies from different manufacturers in various concentrations, but only CD63 and CD9 gave signals at the expected molecular weight on Western blots. Until antibodies specific to equine EV-related proteins become available, we recommend using a combination of methods, including techniques that do not require antibodies, such as NTA and TEM. TEM can discriminate single EVs from similar-sized non-EV particles and is therefore widely used to monitor the quality and purity of EV-containing samples, e.g., for therapeutic application or downstream analysis [53,73,74,75,76].

In addition to a panel of markers, there is a need for more detailed reporting of methodologies and results to facilitate data comparison. For example, the different EV characterisation methods, a dynamic light scattering (NTA) or an antibody (FT-FC)-based technique yield vastly variable particle concentration measurements, as demonstrated by a particles per millilitre count of 8.08 × 10^10^ ± 4.73 × 10^10^ (SEC) and 2.24 × 10^12^ ± 1.6 × 10^12^ (UC) particles measured with NTA versus 35,811 ± 6729 (SEC) and 188,752 ± 71,241 (UC) CMG-positive particles or 8470 ± 1751 (SEC) and 49,616 ± 22,588 (UC) CD81-positive particles measured with FT-FC. NTA measures the size of particles and their concentration based on the physical principle of Brownian motion. In contrast to FT-FC, where lipid dyes or antibodies are used to specifically label EVs, NTA cannot discriminate between contaminations in the form of particles that might derive from external sources such as washing buffers. In addition, the measurements of the concentration and the size distribution are closely linked to the detection limit of the characterisation method, which leads to differences between the measured concentrations [38,41]. Hence, it is paramount to report which platform was used and how measurements were calibrated and normalised.

While the technical aspects of EV isolation and quantification likely encompass the majority of the limitations in EV purity and yield estimation, biological factors, such as EV-producing cell type, cell culture, confluence and stimulation determine EV cargo and hence therapeutic efficacy and may also influence EV yield [77,78]. As the regenerative and immunomodulatory capacity of MSCs decreases with increasing donor age, the use of allogeneic MSCs to produce EVs represents an attractive cell-free off-the-shelf treatment option, which allows the selection of optimal donor cells, scalable, standardised manufacturing and treatment optimisation for the target disease [79,80]. However, extensive culture expansion drives MSCs toward replicative senescence and a consequent decline in quality with diminished immunosuppressive and regenerative capacity and proinflammatory features [81,82]. Indeed long-term in vitro culture (high passage) was recently shown to have a greater effect on MSCs (increased β-Galactosidase level) than the natural ageing process of their donor [82]. Ageing and senescence impact MSC characteristics in several ways and also affect the release of bioactive factors and EVs [80,83]. Interestingly, the secretion of EVs by MSCs increases with donor age and late passage cultures, potentially to dispose of undesirable molecules or as distress signal [83,84]. Furthermore, EVs released by senescent cells contain an altered cargo that may contribute to senescence propagation and chronic inflammation [80,83,85]. Therefore, studies focusing on identifying the optimal EV-producing cells (e.g., MSCs) and culture conditions for therapeutic applications and developing standardised EV manufacturing processes also for the selection, isolation and culture of the EV-producing cells are urgently needed to ensure a consistent EV quality, facilitate the identification and production of effective dose ranges and advance the clinical translation of EV based therapies. In addition, further studies assessing the cargo quality and quantity of EVs isolated using different methods are needed to compare the therapeutic potential of the various EV preparations and establish standardised EV manufacturing processes.

In conclusion, our work highlights the problems resulting from the lack of standardised EV isolation and characterisation methods and establishes MISEV-based criteria for the definition of equine MSCs-derived EVs to address the limited availability of cross-reacting markers for horses using multimodal characterisation techniques, including the expression of CD9, CD63 (Western blot) and CD81 (FT-FC), an EV-appropriate size range (between 30 and 150 nm, FT-FC, NTA and TEM) and the presence of the characteristic lipid bilayers surrounding EVs (TEM).

## 4. Materials and Methods

All experiments were conducted using three biological replicates (MSC derived from 3 horses, *n* = 3) and technical triplicates.

### 4.1. Bone Marrow Collection and MSC Isolation

Bone marrow was harvested from horses (donor 1: 11-year-old Tinker mare; donor 2: 6-year-old Pura Raza Espanola gelding; donor 3: 23-year-old Austrian Warmblood mare) euthanised for reasons unrelated to this study. Based on the “Good Scientific Practice, Ethics in Science and Research regulation” implemented at the University of Veterinary Medicine Vienna, the Institutional Ethics Committee of the University of Veterinary Medicine Vienna and the Austrian Federal Ministry of Educations Science and Research, in vitro cell culture studies do not require approval if the cells were isolated from tissue which was obtained either solely for diagnostic or therapeutic purposes or in the course of other institutionally and nationally approved experiments.

Immediately postmortem, bone marrow was harvested from the sternum under sterile conditions using a Yamshidi bone marrow aspiration needle (TIN3015, CareFusion, San Diego, CA, USA) and a syringe prefilled with heparin 5000 IE/mL (Gilvasan, Vienna, Austria).

The aspirated bone marrow was mixed with 1× PBS with Mg/Ca (PBS +/+, Gibco, Waltham, MA, USA, 14190144) (1:1) and filtered through a 100 µM cell strainer (Greiner Bio-One, Kremsmünster, Austria, 542000). The mononuclear cell fraction was isolated by density gradient centrifugation using Ficoll Paque Premium (GE Healthcare, Chicago, IL, USA, 11743219) as previously published [85,86]. In brief, the collected bone marrow–PBS +/+ mix was layered onto the Ficoll and centrifuged at room temperature for 30 min at 300 g (Hettich, Westphalia, Germany, Rotanta 460R) without brake. The buffy coat was collected. After washing, the obtained mononuclear cells were seeded in DMEM with 1 g/L glucose, L-glutamine, 110 mg/L sodium pyruvate (Gibco, 31885023) supplemented with 10% FCS (Sigma Aldrich, St. Louis, MO, USA, F7524), 1% Pen/Strep (Sigma, St. Louis, MO, USA, P433-100 mL) and 1% amphotericin B (Biochrom, Cambridge, UK, A 2612-50 mL) and cultured in an incubator with 20% O_2_ and 5% CO_2_. In the following days, nonadherent cells were removed by washing and medium change. After three weeks, MSCs were harvested and frozen until further use.

### 4.2. Trilineage Differentiation

Differentiation experiments were carried out in technical triplicates and maintained in culture for three weeks. The media were exchanged every three days. The controls were cultured in DMEM supplemented with 10% FCS, 1% Pen/Strep and 1% amphotericin B. For adipogenic and osteogenic differentiation, 4000 MSCs were seeded per well of a 12-well plate in DMEM supplemented with 10% FCS, 1% Pen/Strep and 1% amphotericin B for 48 h. After washing with PBS +/+, adipogenesis (ThermoFisher, Waltham, MA, USA, A1007001) or osteogenesis (ThermoFisher, A1007201) differentiation media were added. For chondrogenic differentiation, 350,000 MSCs were pelleted per 15 mL Falcon tube and resuspended in chondrogenesis differentiation media (ThermoFisher, A1007101).

### 4.3. Staining Protocols

#### 4.3.1. Oil Red Staining

Six parts of Oil red O (Sigma, O0625-25G) were diluted with four parts aqua dest, mixed overnight at room temperature and filtered. Cells were fixed with 60% isopropanol (Riedel de Haen, Seelze, Germany, 24137) for 5 min and afterwards incubated with the working solution of Oil red O for 10 min. Finally, the differentiated cells were washed with 60% isopropanol and aqua dest, counterstained with haematoxylin (Merck, Kenilworth, NJ, USA, 108562) and washed with aqua dest.

#### 4.3.2. Alcian Blue Staining

Paraffin-embedded samples of chondrocyte pellets were cut using a microtome (CUT2511A, MicroTec, Brixen, Italy). Paraffin blocks were precooled at −15 °C and cut to 3 μm sections. The slices were transferred into cold water using two wet brushes and further transferred to 40 °C warm water with nonadhesive standard microscope slides (Carl Roth, Karlsruhe, Germany). They flattened out and were collected with the super frost adhesive microscope glass slides (Thermo Scientific). The slides were labelled and were left to dry at room temperature overnight. Slides were heated for 20 min in the incubator at 60 °C and submerged in Xylol twice for 10 min at room temperature. Afterwards, the slides were incubated for 5–10 min in 100% isopropanol and decreasing ethanol concentrations (96%, 70%, and 50%) for 5–10 min each. Slides were then washed in aqua dest. and stained in haematoxylin for 3 min. Finally, the slides were washed with aqua dest. and stained with eosin for 30 s. After soaking in ethanol and dehydrating in xylene, slides were mounted with Aquatex mounting medium (Merck Millipore, Burlington, MA, USA) and dried overnight. Slides were analysed with the FL Auto Imaging System (Invitrogen (Waltham, MA, USA), EVOS (Hong Kong, China), Thermo Scientific).

#### 4.3.3. Von Kossa Stain

Cells were fixed with 5% formol (Sigma) incubated with 5% silver nitrate (Roth, 9370.9) for 20 min under UV light and washed with aqua dest. Afterwards, they were fixed with 5% sodium thiosulfate, washed with aqua dest. and stained with real red (Waldeck, 221833) for 10 min and washed again.

### 4.4. EV Isolation

MSCs were thawed and expanded in chemically defined complete medium (DMEM) supplemented with 10% FCS (Sigma Aldrich, F7524), 1% Pen/Strep and 1% amphotericin B until 80–90% confluency, passaged with trypsin (Gibco, 25300-096) and seeded at a defined density of 4 × 10^6^ cells per T175 flask (Sarstedt, Nümbrecht, Germany, 833912002). All cells used were at passage 2, cell morphology was assessed daily, and viability was measured using Trypan blue dye exclusion in conjunction with an automated cell counter (ThermoFisher Countess 3). After 24 h, cells were washed twice with 10 mL filtered (0.22 µm filter (Sarstedt, 831822)) PBS +/+ and then cultured in 22 mL serum-free DMEM (plus 1% Pen/Strep, 1% amphotericin). After 48 h and doubling of the cell number, which was estimated from preliminary studies with a population doubling level (PDL) of 3 (PDL = 2 + 3.322 (log 8 × 10^6^–log 4 × 10^6^)), the conditioned medium was collected and transferred into 50 mL Falcon tubes (Sarstedt, 64547254) and precentrifuged at 3000× *g* for 20 min at 4 °C to remove undesired cell debris. The obtained supernatant (SN) was carefully collected without disturbing the cell debris pellet and immediately processed.

#### 4.4.1. Sequential Ultracentrifugation

For ultracentrifugation, 10 mL of precentrifuged SN was transferred into ultracentrifuge tubes (Beranek, 5047) and spun in an ultracentrifuge (L-100K, Beckman coulter, Brea, CA, USA) using a 55.2 Ti fixed-angle rotor at 100,000× *g* (35,000 rpm) for 1.5 h at 4 °C. The invisible pellet was recovered by scraping the walls of the tube where the pellet was suspected to be and simultaneously aspirating with a 5 mL pipette. Together with 1 mL SN, the pellet was transferred into 1.5 mL ultracentrifuge tubes (Beckman coulter, 357448). The second ultracentrifugation was performed in a tabletop ultracentrifuge (Optima Max, Beckman coulter) using a TLA 45 fixed-angle rotor at 100,000× *g* (45,000 rpm) for 1.5 h at 4 °C. Finally, the SN was aspirated and discarded. The EVs were resuspended and either processed for experimental use immediately or stored at −80 °C. The solution for the resuspension of the pellet was chosen according to the requirements for the follow-up experiments. (HEPES (Invitrogen, 14291DJ) for staining or storage, RIPA buffer (Sigma, R0278-50 mL) for Western blot, medium or filtered PBS for treatment experiments).

#### 4.4.2. Size Exclusion Chromatography (SEC)

According to the manufacturer’s protocol, EVs were isolated using qEV10/35 nm (IZON, qEV10 35 nm) columns and the Automated Fraction Collector (IZON). All reagents (IZON columns and filtered PBS) were stored at 4 °C and warmed to room temperature before starting the separation. A size exclusion column was mounted into the Automated Fraction Collector (AFC) with the reservoir attached on top, programmed to collect the desired fractions and flushed with 120 mL filtered PBS +/+. All PBS residues were removed with a pipette, and 10 mL of precentrifuged SN was loaded into the reservoir. The AFC has an integrated scale that automatically collects the void volume (20 mL) and the single fractions (5 mL per fraction). SN was loaded into the frit, and the reservoir was filled with filtered PBS +/+. Fraction 1 was discarded, and fractions 2 and 3, containing the desired EVs, were either directly processed or stored at −20 °C. The column was flushed with 120 mL sodium azide (Sigma, RTC000068-1L) and stored for further use at 4 °C.

### 4.5. Nanotracking Analysis

According to the manufacturer’s protocol, the samples were diluted in filtered PBS +/+ (1:1000) and loaded into the device. Samples were measured in scatter mode with a 488 nm laser. Minimum brightness was set to 30, the minimum area to 10, maximum area to 1000, maximum brightness to 255, shutter to 400 and temperature to 25 °C. Isolates were measured in technical and biological triplicates.

### 4.6. Flow Cytometry

#### 4.6.1. MSCs

For the flow cytometry, cells were trypsinised at passage 2, and 1 × 10^5^ cells per sample were washed with PBS +/+ supplemented with 2% FSC (Sigma Aldrich, F7524). The following monoclonal antibodies and respective isotype controls were used for the flow cytometry: PE-CD29 (Clone TS2/16, Mouse IgG, 1:50, Biolegend, San Diego, CA, USA), FITC-CD31 (Clone CO.3E1D4, IgG2a, 1:50, Biorad, Hercules, CA, USA), FITC-CD44 (Clone 25.32, IgG, 1:50, Biorad), Purified CD90 (Clone DH24A, IgM, 1:50, Monoclonal Antibody Center), FITC-PanB cells (Clone CVS36, IgG1, 1:50, Biorad). A total of 1 × 10^4^ events were measured per sample.

#### 4.6.2. EVs (Fluorescence-Triggered Flow Cytometry (FT-FC))

Size distribution was evaluated by measuring the CD81 signal and the CellMask Green plasma membrane (CMG) signal separately. EVs were either isolated using UC or SEC as described above. The CMG staining and the gating strategy were performed as described before [60]. The SSC-based size gates were set according to the 100, 200 and 500 nm FITC-labelled silica beads and were merged to a small (≤200 nm), intermediate (>200–<500 nm) and large EV (≥500 nm) size range gate.

### 4.7. Western Blot

#### 4.7.1. Protein Extraction

Cells were cultured in 10% DMEM until they were 70% confluent. A lysis buffer consisting of RIPA buffer (50 mL) and 2% protease inhibitor cocktail (Roche, Basel, Switzerland, 04693124001) was added to the plates, and the cells were scraped off the plate using cell scrapers (Greiner, 541070). After spinning with 17,200 *g* at 4 °C for 20 min, SN was transferred to an Eppendorf tube and stored at −20 °C. The EV pellet was resuspended in 75 μL of HEPES, vortexed at high speed and stored at −80 °C or directly processed for Western blotting. To determine the protein concentration, a BCA protein assay kit (Thermo Fisher 23227) and the Qubit protein kit (Invitrogen, Q33211) were used according to the manufacturer’s protocol.

#### 4.7.2. Western Blotting 

A 10× electrophoresis buffer (running buffer) was prepared by mixing 30.3 g/L Tris (Roth, 0188.3), 142.6 g/L glycine (Roth, 0079.3), 1% SDS (Roth, 0601.1) and filling up the volume to 1 L with ddH2O. For working conditions, the stock solution was diluted 1:10 with ddH2O. Harlow buffer (transfer buffer) consists of 80 mL 10× electrophoresis buffer, 200 mL methanol (Roth, 0082.3), filled up to 1 L with ddH2O and stored at 4 °C. Then, 10× TBS was produced by adding 87.66 g/L NaCl (Roth, 0601.1) to 60.57 g/L Tris and filling up to 1 L with ddH2O before adjusting the pH to 7.4 with 6 M HCl (Merck, 1101641000). TBS/T was prepared by diluting 10× TBS 1:10 in ddH2O and adding 1 mL/L of Tween 20 (Roth, 27.2).

A total of 25 μg from the UC isolates of each sample was mixed with Laemmli 2× concentrate (Sigma S3401-1VL) nonreducing (without beta-mercaptoethanol for CD9 and CD63) and filled up to a certain amount with ddH2O, which depends on the pocket size of the precast gel (BioRad, 4561093) (15 comb: 10 μL and 10 comb: 20 μL). After denaturing for 10 min at 70 °C, the samples were loaded on the gel with 5 μL protein marker (Thermo Fisher, 26619). The power supply was set to constant 90 V for 10 min and was afterwards increased to constant 120 V for 70 min. To transfer the protein on a PVDF membrane (BioRad, 1620177), wet blotting using two Whatman papers (BioRad, 1703932) facing the cathode and the PVDF membrane (which was soaked in methanol before use) in between the gel and two Whatman papers facing the anode was performed. All the materials, including the sponges placed at the two ends of the sandwich, were soaked in Harlow buffer, and the blotting procedure was carried out in a tank containing an ice block at constant 350 mA for 1 h. After the blotting process, it was essential to mark the side of the membrane facing the gel. The membrane was washed 3× in TBS/T and blocked for 1 h with 5% nonfatty milk (Maresi) TBS/T buffer. Before incubating the membrane overnight with the first antibody (CD9 antihuman, (BioVision, Milpitas, CA, USA, 6938) or CD63 antihuman (SBI, EXOAB-CD63A-1)) on a shaker at 4 °C, and the membrane was again washed 3× in TBS/T. Finally, the membrane was washed 3× in TBS/T and incubated for 1 h with HRP-linked heavy and light chain antibodies. A Super Signal West Femto (Thermo Scientific, 34094) protein detection kit was used for the detection. The membrane was developed in an imaging system (BioRad, ChemiDoc).

#### 4.7.3. Membrane Stripping

The buffer for the stripping procedure consisted of 15 g glycine (VWR, Radnor, PA, USA), 1 g SDS (Sigma), 10 mL Tween 20 (Sigma) and filled up to a total volume of 1 L with dH2O prior to adjusting the pH to 2.2. The membrane was incubated twice for 5–10 min with the stripping buffer, washed twice for 10 min in PBS and washed twice for 5 min with TBS/T. Afterwards, the membrane was ready for the blocking step.

### 4.8. Transelectron Microscopy

EVs were isolated from the same flask. After SEC, the isolated EVs were pelleted using a tabletop ultracentrifuge and embedded in paraffin. The block was cut (70 nm), contrasted with Uranyl acetate and lead citrate and examined with an electron light microscope (EM 900, Zeiss, Jena, Germany) using a slow-scan CCD 2K- wide-angle dual-speed camera (TRS).

### 4.9. Statistical Analysis

The statistical analysis was performed using Graph Pad Prism v.6.01 (GraphPad Software, San Diego, CA, USA). For the comparison of two normally distributed samples, the Student’s *t*-test was conducted. The number of used donors (*n*), the *p*-values and the respective statistical significance are indicated in each figure. The data are plotted as mean ± standard deviation.

## Data Availability

The data presented in this study are available on request from the corresponding author.
